# The association between biliary tract inflammation and risk of digestive system cancers: A population-based cohort study

**DOI:** 10.1097/MD.0000000000004427

**Published:** 2016-08-07

**Authors:** Tsung-Yu Tsai, Che-Chen Lin, Cheng-Yuan Peng, Wen-Hsin Huang, Wen-Pang Su, Shih-Wei Lai, Hsuan-Ju Chen, Hsueh-Chou Lai

**Affiliations:** a Graduate Institute of Clinical Medical Science; b School of Medicine; c Division of Hepato-gastroenterology, Department of Internal Medicine; d Management Office for Health Data; e Department of Public Health; f Department of Family Medicine; g School of Chinese Medicine, China Medical University, Taichung, Taiwan.

**Keywords:** colon cancer, gastrointestinal intestinal cancer, inflammation, pancreas

## Abstract

The relationship between biliary tract inflammation (BTI) and digestive system cancers is unclear. This study aimed to evaluate the association between BTI and the risks of digestive system cancers.

Using the Taiwan National Health Insurance claims data, information on a cohort of patients diagnosed with BTI (n = 4398) between 2000 and 2009 was collected. A comparison cohort of sex-, age-, and index year-matched persons without BTI (n = 17,592) was selected from the same database. The disease was defined by the ICD-9-CM. Both cohorts were followed until the end of 2010 and incidences of digestive system cancers were calculated.

The results revealed an increase in adjusted hazard ratio (aHR) of biliary tract cancer (24.45; 95% confidence interval [CI]: 9.20–65.02), primary liver cancer (1.53; 95% CI: 1.07–2.18), and pancreatic cancer (3.10; 95% CI: 1.20–8.03) in patients with both gallbladder and BTI. The aHR of stomach cancer was also found to be increased (2.73; 95% CI: 1.28–5.81) in patients with gallbladder inflammation only. There were no differences in esophageal cancer (aHR: 0.82; 95% CI: 0.23–2.87) and colorectal cancer (aHR: 0.92; 95% CI: 0.59–1.45). The aHR for digestive system cancers increased by 3.66 times (95% CI: 2.50–5.35) and 12.20 times (95% CI: 8.66–17.17) in BTI visits frequency averaged 2 to 4 visits per year and frequency averaged ≥5 visits per year, respectively.

Patients with BTI have significantly higher risk of digestive system cancers, particularly biliary tract, pancreatic, and primary liver cancers, compared with those who are without it.

## 1 Introduction

Inflammation increases cancer risk because immune and inflammatory components induce tumorigenesis.^[1]^ Biliary tract inflammation (BTI) is a common illness that includes cholecystitis and cholangitis. It may induce bacteremia and is associated with high morbidity and mortality. The most common infecting organisms are *Escherichia coli*, *Enterobacteriaceae* spp, and *Klebsiella pneumoniae* ascending from the gastrointestinal (GI) tract.^[2,3]^ Even with available treatments via endoscopic retrograde cholangiopancreatography, percutaneous drainage, and surgery, mortality is still as high as 11%.^[4]^

Digestive system cancers are common worldwide. These include esophageal, gastric, colorectal, small intestinal, biliary tract, pancreatic, and primary liver cancers. Different populations have different incidence, often influenced by sex, races, and residence. The incidence has also increased because of improvements in diagnostic techniques and genetic technology.^[5]^ Environmental or occupational exposure to toxic substances with carcinogenic potential effect has also increased the risk of GI tract cancer such as hepatocellular cancer.^[6]^ The relationship between hepato-BTI and digestive system cancers has been reported in the literature. For instance, patients with cholelithiasis or higher body mass index (BMI) may have higher risk of biliary tract cancer.^[7]^ Patients with pyogenic liver abscess (PLA) also have higher risk of GI tract cancers.^[8]^ Cholelithiasis or choledocholithiasis may increase the risk of pancreaticobiliary cancer, but not extra-pancreaticobiliary cancer.^[9]^ Others report that BTI may increase the risk of colorectal cancer.^[10]^ On the basis of cell line and clinical study, Lu and Mack^[11]^ supposed that inflammation in biliary tract would stimulate cholangiocyte toll-like receptor promotion of the ongoing inflammatory response. It also induces gene deficiencies in the susceptibility to immune-mediated cholangiopathies, which might easily induce cancer.^[11,12]^

In the past years, there had been no large-scale, population-based study exploring the relationship between BTI and the risk of digestive system cancers. Thus, this study aimed to investigate the risk of digestive system cancers among BTI patients with at least 1-year follow-up after the diagnosis of BTI, compared with patients without BTI during the same period.

## 2 Methods

### 2.1 Data source

In 1996, the Taiwan government organized a national-wide and single-payer health insurance program called the “Taiwan National Health Insurance Program” (Taiwan NHIP). Since 1998, the NHIP had covered >99% of the 23 million Taiwanese.^[13]^ Consequently, the National Health Research Institutes (NHRI) established the National Health Insurance Research Database (NHIRD) composed of the annual reimbursement claims data from the NHIP.

The present study used the Longitudinal Health Insurance Database (LHID), a subset of NHIRD, to construct the study population. The LHID contained the annual reimbursement claims data from 1 million insured people randomly selected in 1996 to 2000. Based on the NHRI report, the distribution of age and sex ratios between LHID and NHIRD was not different. The NHRI provided a scrambled and anonymous identification number for each insured person to link each claim data, including registry for beneficiaries, out-patient and in-patient visits, and other medical services records. The Institutional Review Board of China Medical University in central Taiwan exempted this study from the informed consent requirement (CMU-REC-101-012).

The disease record in NHIRD was defined by the disease according to the International Classification of Diseases, Ninth Revision, Clinical Modification (ICD-9-CM). Cancer diagnosis database was collected from the catastrophic illness registry and the history of other diseases was gathered from in-patient and out-patient files. The Registry of Catastrophic Illness Database is a subpart of the NHIRD. In Taiwan, insured people with major diseases can apply for a catastrophic illness certificate that grants exemption from copayment. The issuance of catastrophic illness certificates was validated by at least 2 specialists, based on careful evaluation of the medical records, laboratory studies, and imaging studies. Individuals would be issued a catastrophic illness certificate when they meet the diagnostic criteria for major diseases. Cancers, cirrhosis with esophageal varices bleeding, uremia on hemodialysis, and organ failure are statutorily included in the catastrophic illness category.

### 2.2 Study population

This study was a population-based retrospective cohort study design. The cohort of patients with BTI consisted of newly diagnosed BTI (ICD-9-CM 575.0, 575.1, 575.2, 576.1, and 576.2) cases between 2000 and 2009. The date of diagnosis was set as the index date of BTI. The BTI cohort was separated on the basis of the inflammatory location in the observation time: individuals with only gallbladder inflammation (ICD-9-CM 575.0, 575.1, and 575.2); individuals with only BTI (ICD-9-CM 576.1 and 576.2); and individual with both inflammations.^[10]^

The comparison cohort was established by age (per 5 years), sex, and diabetes mellitus (DM) (ICD-9-CM 250.00–250.93) history frequency matched at a 1:4 ratio from individuals without BTI diagnosed in the LHID. The index date of the comparison cohort was randomly assigned a day and month within the same year of the index date of the patient in the BTI cohort with whom they were matched. Follow-up of the 2 cohorts began 6 months after the index date. Individuals with cancer (ICD-9-CM 140–208) diagnosed before the index date or any cancer occurrence before the follow-up started were excluded. The follow-up was terminated when the patient quit the insurance (including fulfilling mandatory military service, expatriated to other country, imprisonment, death, etc.), when digestive system cancers developed, or until December 31, 2010 (end of the study).

The main study outcome was the occurrence of digestive system cancers (ICD-9-CM 150–157). The influence of BTI on the subtype of digestive system cancer risk was also investigated. Digestive system cancers were categorized by location: esophageal cancer (ICD-9-CM 150), stomach cancer (ICD-9-CM 151), small intestine cancer (ICD-9-CM 152), colorectal cancer (ICD-9-CM 153 and 154), biliary tract cancer (ICD-9-CM 156), primary liver cancer (ICD-9-CM 155), and pancreatic cancer (ICD-9-CM 157).

The socio-demographic factors included age, sex, and occupational status. The occupational status includes government, school, private enterprise, occupational member, farmer and fishermen, low-income household, and veterans. Comorbidities associated with digestive system cancers were also collected as confounding factors. Comorbidities were included during diagnoses before the index date. These included PLA (ICD-9-CM 572.0), pancreatitis (ICD-9-CM 577.0 and 577.1), hepatitis B virus infection (HBVI; ICD-9-CM 070.2, 070.3 and V02.61), hepatitis C virus infection (HCVI; ICD-9-CM 070.41, 070.44, 070.51, 070.54 and V02.62), unspecified chronic hepatitis (UCH; 070.9, 571.4, 571.8 and 571.9), alcoholic liver disease (ALD; ICD-9-CM 571.0–571.3), liver cirrhosis (ICD-9-CM 571.5 and 571.6), and cholelithiasis (ICD-9-CM 574).

### 2.3 Statistical analysis

The distribution of the demographic characteristic and disease history between the 2 cohorts were demonstrated according to the mean and standard deviation (SD) for age and the number and proportion for sex, occupational status, and disease history. The *t* test was used for age and χ^2^ test for categorical variables to assess the difference between the 2 study cohorts. The incidence of developing digestive system cancers was calculated as the total number of digestive system cancer occurrences divided by the total sum of observation time in each groups by 10,000 person-years. The Kaplan–Meier method was used to measure the cumulative digestive system cancer incidence curves and the log rank test was used to assess the difference of these curves. The risk of developing digestive system cancer differed between individuals with and those without BTI, as estimated by the multivariable Cox proportional hazard model and was presented by hazard ratios (HRs) and 95% confidence intervals (CIs).

All data management and statistical analysis were performed using SAS 9.3 software (SAS Institute, Inc., Cary, NC). The R software (R Foundation for Statistical Computing, Vienna, Austria) was used to draw the cumulative incidence curves. Significance was set at *P* <0.05.

## 3 Results

**Figure 1:**
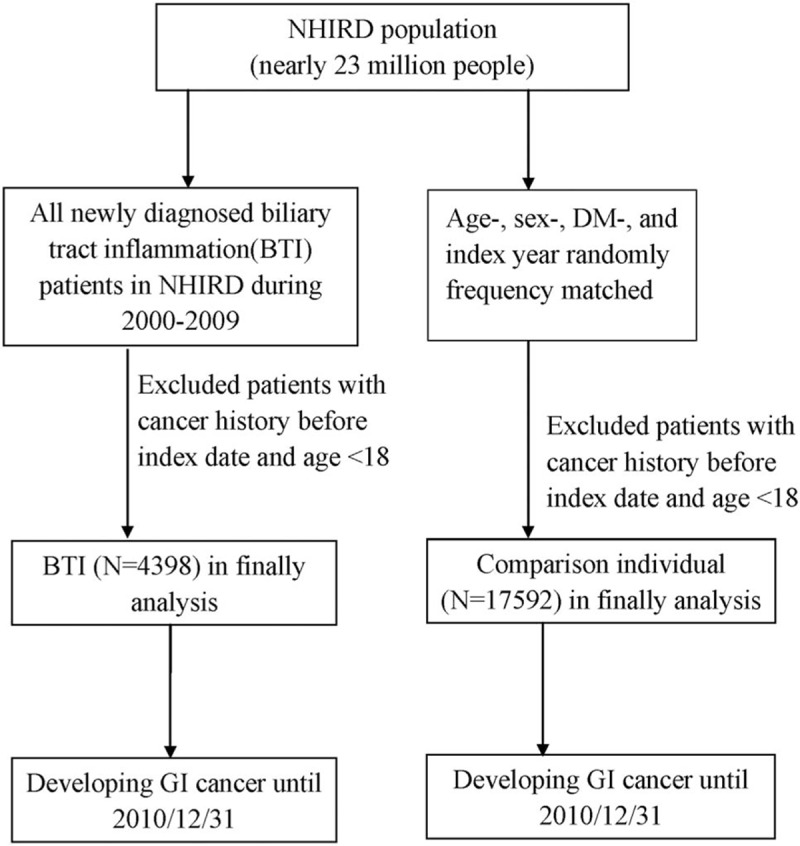
Flow chart presenting study subjects.

In this study, there were 4398 individuals with BTI and 17,592 individuals without BTI (Fig. 1). The 2 cohorts had similar mean age (58 years), sex ratio (female, 50%), and proportion of DM history (Table 1). Individuals with BTI had a higher prevalence of engaging in farmers, fishermen occupations, than those without BTI.

**Table 1:**
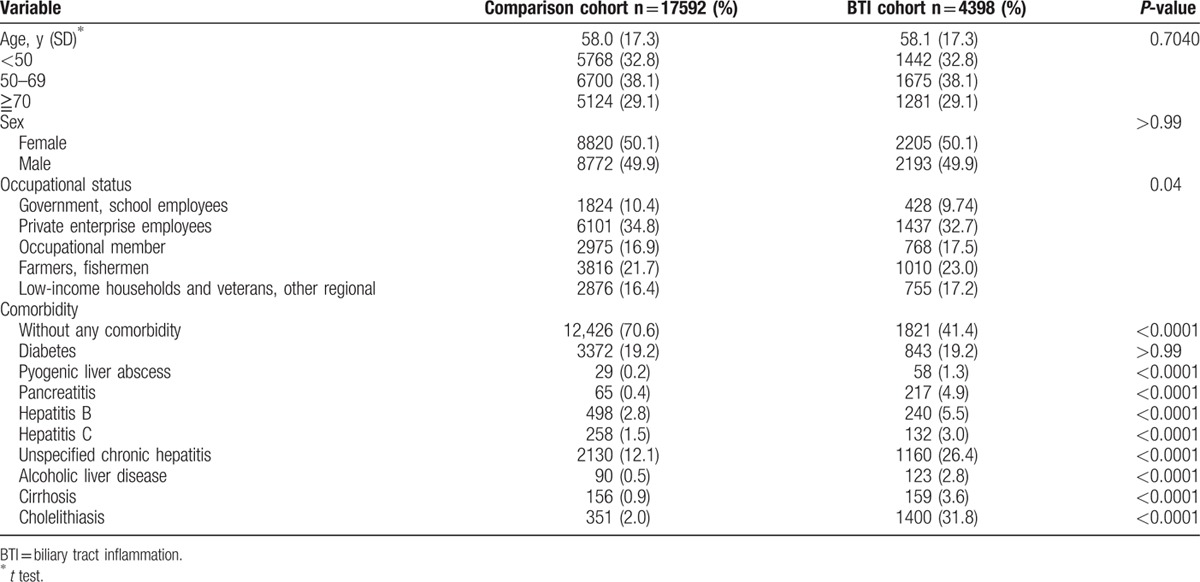
Baseline demographic status and co-morbidity compared between the biliary tract infection and comparison cohort.

**Figure 2:**
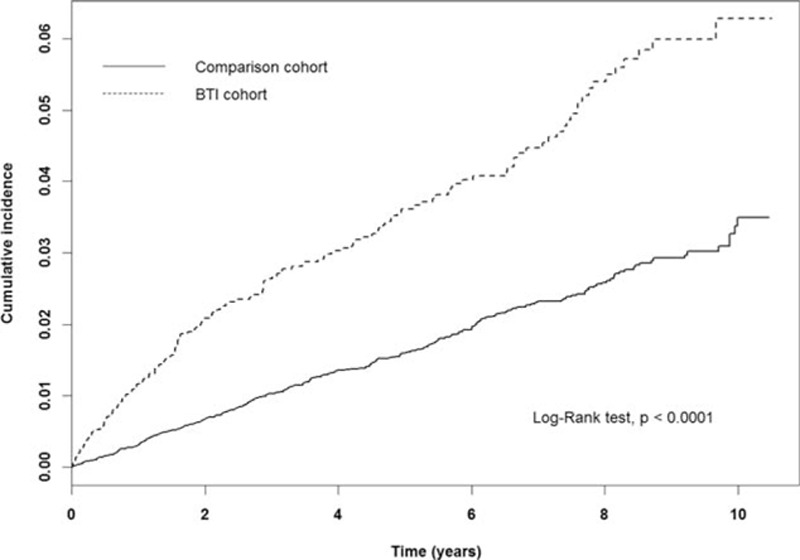
Cumulative incidence of digestive system cancer in the study population.

The cumulative incidence of developing digestive system cancers in the BTI cohort was 72.99 per 10,000 person-years and 34.40 per 10,000 person-years in the comparison cohort (Table 2). The cumulative incidence curve of developing digestive system cancer in individuals with BTI was significantly greater than the incidence curve in those without BTI (*P* <0.0001, by the log-rank test) (Fig. 2). After adjustments for age, sex, occupational status, DM, PLA, pancreatitis, hepatitis B infection, hepatitis C infection, unspecified chronic hepatitis, alcoholic liver disease, cirrhosis, and cholelithiasis, individuals with BTI had a 1.68-fold increased risk than those without BTI (adjusted hazard ratio [aHR]: 1.68, 95% CI: 1.35–2.09). Individuals with only gallbladder inflammation might have a trend of increasing risk of developing digestive system cancers but did not reach statistical significance (aHR: 1.14, 95% CI: 0.84–1.57). However, individuals with only biliary inflammation or with both subtypes of BTI were still significantly associated with increased risk of developing digestive system cancers. (aHR: 2.13 and 2.37, respectively).

**Table 2:**
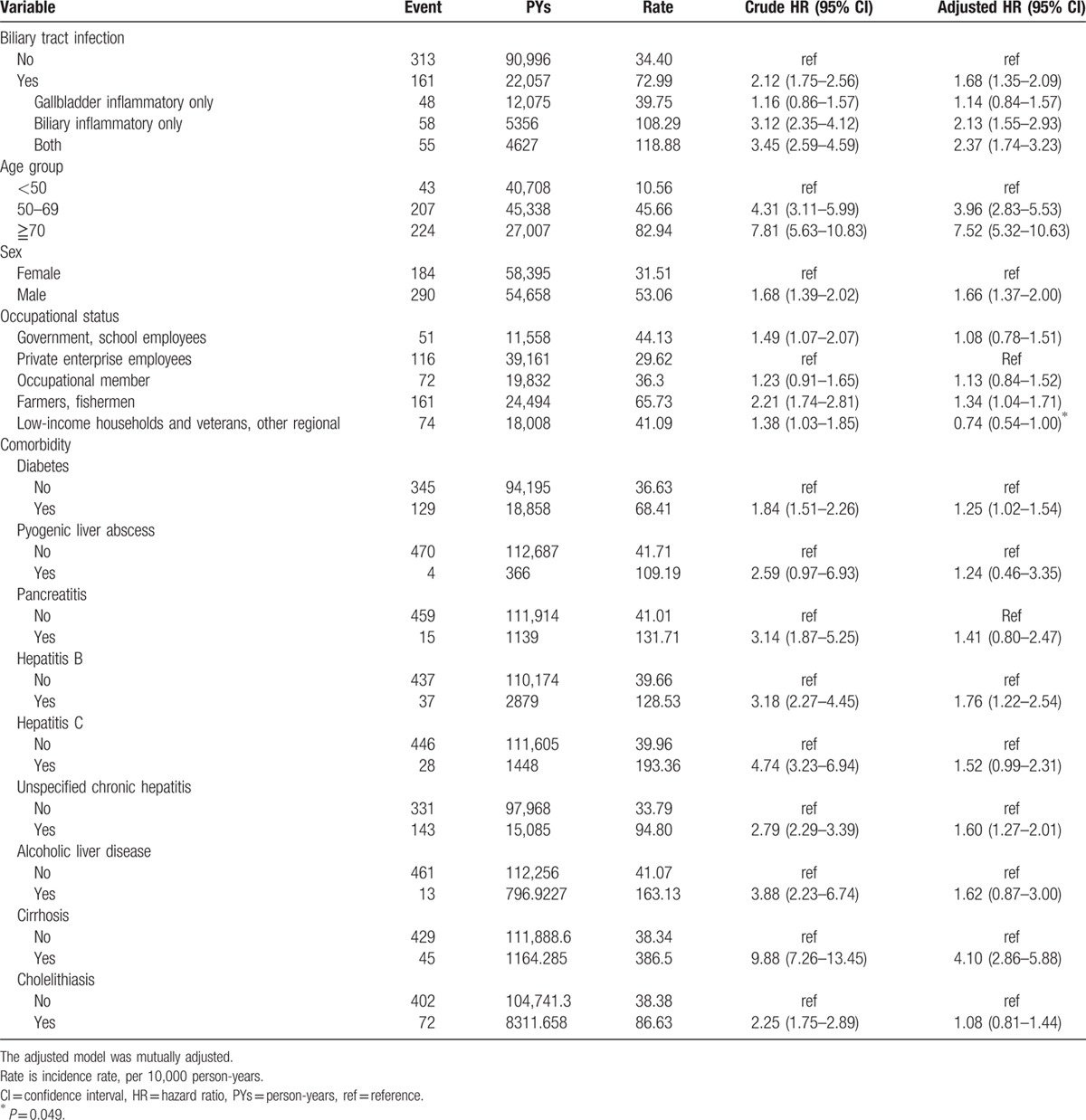
The incidence of digestive system cancer and stratified analysis by multivariate Cox proportional hazards regression analysis measured the hazard ratio for the study cohort.

The risk of different subtype of digestive system cancers between the individual with and those without BTI (Table 3) revealed that the main effect of BTI significantly influenced the risk of developing biliary cancer (aHR: 24.45, 95% CI: 9.20–65.02), primary liver cancer (aHR: 1.53, 95% CI: 1.07–2.18), and pancreatic cancer (aHR: 3.10, 95% CI: 1.20–8.03). Although the main effect of BTI was not significantly associated with increased risk of developing stomach cancer (aHR: 1.63, 95% CI: 0.81–3.26), individuals with gallbladder inflammation only had a significant 2.73-fold increased risk of developing cancer, compared with individuals without BTI (aHR: 2.73, 95% CI: 1.28–5.81).

**Table 3:**
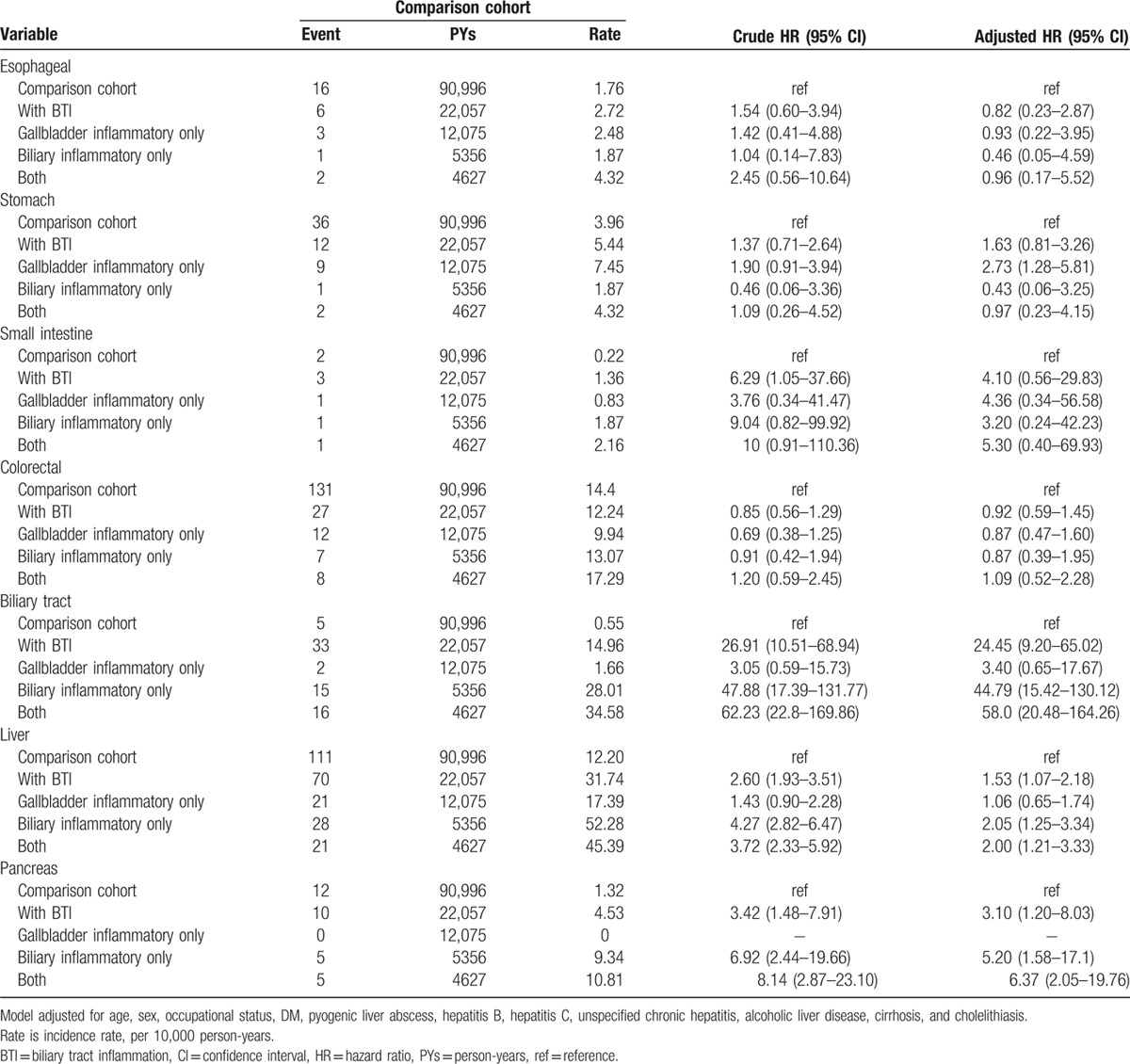
Incidence of different type of digestive system cancer and multivariate Cox proportional hazards regression analysis measured for hazard ratio of the study cohort.

According to the average frequency of BTI visits, the risk of developing digestive system cancers had a significantly increasing trend, with growing frequency of BTI visits (*P* <0.0001) (Table 4). Relative to individuals without BTI, the highest level of BTI visits frequency averaged ≥5 visits per year and even had a dramatic increase in the risk of developing digestive system cancers (aHR: 12.20, 95% CI: 8.66–17.17).

**Table 4:**
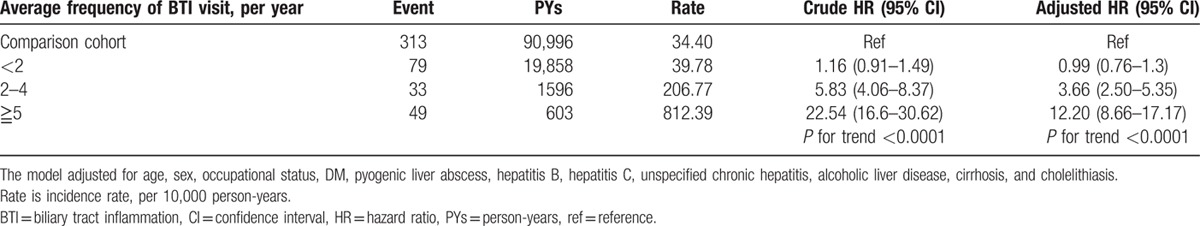
Incidence of digestive system cancer and multivariate Cox proportional hazards regression analysis of HR for the study cohort, by average frequencies of BTI visit.

## 4 Discussion

This is the first large-scale population-based study on the significant association between BTI and digestive system cancers (aHR: 1.68; 95% CI: 1.35–2.09). This study indicates that BTI, including cholangitis and cholecystitis, may significantly increase the risk of biliary tract cancer (aHR: 24.45; 95% CI: 9.20–65.02), pancreatic cancer (aHR: 3.10; 95% CI: 1.20–8.03), stomach cancer in patients with only gallbladder inflammation (aHR: 2.73; 95% CI: 1.28–5.81), and primary liver cancer (aHR: 1.53; 95% CI: 1.07–2.18). The cumulative incidence curve also show an increasing risk of digestive system cancers in BTI patients over time (*P* < 0.0001).

For occupational status, farmer or fisherman had higher risk to develop GI tract cancer; this may be because farmer had higher risk of being exposed to toxic effect such as pesticide which will increase the risk of GI tract cancer. Low-income households and veterans occupation seem to decrease the risk of GI tract cancer (*P* = 0.049), they may be underestimate of the risk of cancer because of their economy status they cannot afford to receive health examination. However, in our NHIRD, occupational status is not detailed registered and toxic substance was seldom diagnosed in Taiwan, this will be our limitation.

Esophageal cancer is the 6th leading cause of cancer deaths in American and adenocarcinoma is the predominate type and Barrett's esophagus is 1 of the risk factors.^[14,15]^ Bernstein et al^[16]^ report that bile acid reflux may induce cell apoptosis in Barrett's esophageal cell line and is the risk factor of adenocarcinoma in esophageal cancer. However, esophageal cancer occurs 20 to 30 times higher in China than in the United States, with squamous cell carcinoma as the predominant type. Environment and diet comes from profound differences in incidence observed in various parts of the world.^[15,17]^ In this study, BTI patients do not have increased risk of esophageal cancer, which may be because of less carcinogenesis effects in bile reflux and remote inflammatory factor of BTI.

Gastric cancer is the 8th most common cancer in Taiwan.^[18]^ Stomach cancer incidence and death rates are twice as high in Asian Americans/Pacific Islanders as in whites.^[19]^ Several studies in cell culture and animal have shown that different kinds of bile acids like taurochenodeoxycholic acid may increase the production of reactive oxygen species (ROS) in cultured gastric carcinoma cells, whereas deoxycholic acid may induce apoptosis in cultured human gastric epithelial cells.^[20,21]^ In the clinical setting, gastric reflux is a risk factor of gastric cancer.^[22]^ The present study shows that the increased risk of gastric cancer in BTI patients is only related to gallbladder inflammation, meaning that the gastric reflux fluid that induces gastric cancer is not by bile acid or a component of bile acid changed by the high acidity of gastric fluid. Inflammation-related cytokines, chemokines, reactive oxygen species, and nitrogen may participate in tumor promotion.^[1]^

Biliary tract cancers, including cancers of the extra-hepatic bile duct, Ampulla of Vater, and gallbladder, are uncommon but highly fatal.^[23,24]^ Sattar et al^[25]^ have found that patients with cholelithiasis have infected bile juice in a study with 100 patients. Ishiguro et al^[7]^ discovered that the risk of biliary tract cancer increased with cholelithiasis (HR: 2.53; 95% CI: 1.56–4.12) in a large-scale study in Japan. New markers such as cytokeratin 19 (CYFRA 21-1), mucins, tumor markers_2_ pyruvate-kinase (TuM_2_ PK), and metalloproteinase-7 (MMP-7) have helped to diagnose cholangiocarcinoma.^[26]^ The present study reveals that patients with BTI have increased risk of biliary tract cancer (aHR: 24.45; 95% CI: 9.20–65.02) but BTI patients with gallbladder inflammation had no increased risk (aHR: 3.40; 95% CI: 0.65–17.67). Gallstone is the most common etiology (90%–95%) that induces gallbladder inflammation and can be successfully treated by cholecystectomy. However, BTI has more complicated etiology and high recurrence rate (7%–47%) and chance for becoming chronic inflammation.^[27]^ The study showed lower HR of biliary tract cancer incidence in gallbladder inflammation compared with BTI. This might be because of the inflammation process of gallbladder that could be stopped by cholecystectomy and in BTI, there is no sufficient way to stop the inflammation. Thus, BTI may increase the risk of biliary tract cancer by increasing risk of carcinogenesis and local inflammation.

Primary liver cancer, including hepatocellular carcinoma, and intra-hepatic cholangiocarcinoma, is the second most common cancer in Taiwan.^[18,28]^ Chronic hepatitis B and hepatitis C are the most well-known carcinogenic sources of primary liver cancer. DM is a systemic chronic inflammation disease associated with increased risk of primary liver cancer.^[29]^ Biondi et al^[30]^ investigated that the molecular marker, chromogranin A, maybe a useful marker for early diagnosis of HCC.^[31]^ This study shows that patients with BTI may have increased risk of liver cancer (aHR: 1.53; 95% CI: 1.07–2.18). This result is consistent with those of Razumilava et al^[32]^ who reported that primary sclerosing cholangitis increased the risk of HCC and cholangiocarcinoma.

Pancreatic cancer is associated with a high mortality rate and is 1 of the top 5 causes of death from cancer.^[33]^ This study shows that BTI patients may increase the risk of the pancreatic cancer (aHR: 3.10; 95% CI: 1.20–8.03). Adachi et al^[34]^ had investigated the bile reflux into the pancreatic duct, which was shown to induce development of intra-ductal papillary carcinomas of the pancreas in a hamster surgical model. Lai et al^[35]^ showed that DM patients with gallstones or cholecystitis increase the risk of pancreatic cancer. Thus, inflammation is related to pancreatic cancer.^[1,36]^

Small bowel cancer is <5% in GI tract cancers and has mildly increased in recent years,^[19,36]^ with 50% of adenocarcinomas of the small intestine in the duodenum.^[37]^ An animal study has shown that unconjugated bile acids contribute to peri-ampullary tumor formation in the setting of an *Apc* mutation.^[38]^ This study reveals that BTI patients do not have increased risk of small bowel cancer. This may be because of the small population of small bowel cancer or the short duration of follow-up.

Colon cancer is the 3rd leading cause of cancer-related death in Taiwan in 2008. Some inflammatory diseases like systemic chronic inflammation of Type 2 DM^[39]^ and local inflammation of ulcerative colitis^[40]^ are associated with increased risk of colorectal cancer. Uccello et al^[41]^ indicate that probiotics may reduce colon cancer risk, alter intestinal microflora, inactivate of cancerogenic compounds, and compete with pathogen microbiota. Lin et al^[10]^ have reported that colorectal cancer for those with bile duct inflammation is 3.30 times (95% CI: 1.87–5.84) as high as for those without BTI within the 5-year follow-up period. There is no increased risk of colorectal cancer with gallbladder inflammation. Bile acids can induce DNA damage and apoptosis in colon cells.^[16]^ However, this study does not show that BTI patients increase the risk of colon cancer. The reasons for the different results may be that this study matched the sample size of DM subjects between BTI patients and a comparison cohort.

In Witjes et al, Nienhuijs et al, and Dassen et al's large-scale population studies in the Netherlands from 1989 to 2008, the incidence of gastric and gallbladder cancer had decreased, and the incidence of pancreatic cancer was the same.^[42–44]^ In the present study, we had increased incidence of gastric cancer, gallbladder cancer, and pancreatic cancer in BTI patients. Difference of genetic events in species, diet habit, and can cause the difference result in cancer epidemiology. Also, the health care policy for cancer detection in Taiwan made the disease detection rate higher than before, which makes the incidence of cancer higher than before.

The strength of the present study is its large, representative, nationwide, population-based sample for observing the risk of digestive system cancer in patients with BTI. This large sample size allows a stratified analysis to observe comorbidities that coexist in patients with BTI. However, this study has several limitations. First, detailed information related to the risk of digestive system cancer, such as data on body mass index, high-fat diet, high salt intake, lower physical activity lifestyle, family history of digestive cancer, and consumption of betel nut, smoking and alcohol, is not available. Second, the environmental and occupational exposure to toxic substance was hard to investigate because the diagnosis of toxin was seldom made in Taiwan and occupational status was not detailed registered in our NHIRD. Third, microbiological data are incompletely coded, thereby compromising the relationship of the risk of digestive system cancer. Fourth, generalization of the findings to Western or non-Taiwanese populations is a concern.

In conclusion, in this population-base cohort study, patients with BTI have an increased risk of digestive system cancers compared with a comparison cohort, particularly biliary tract cancer, followed by pancreatic cancer, primary liver cancer, and stomach cancer in patients with gallbladder inflammation only. Further survey of malignancies of the digestive system is suggested in the follow-up of BTI patients.

## Acknowledgment

The authors thank the National Health Research Institute of Taiwan for the use of the NHIRD.
